# Effects of Alkaline Extraction pH on Amino Acid Compositions, Protein Secondary Structures, Thermal Stability, and Functionalities of Brewer’s Spent Grain Proteins

**DOI:** 10.3390/ijms25126369

**Published:** 2024-06-08

**Authors:** Kunn Hadinoto, Jordy Kim-Ung Ling, Siyu Pu, The-Thien Tran

**Affiliations:** School of Chemistry, Chemical Engineering and Biotechnology, Nanyang Technological University, Singapore 637459, Singapore

**Keywords:** biological macromolecules, alternative proteins, protein extraction, alkaline extraction

## Abstract

A high alkaline pH was previously demonstrated to enhance the extraction yield of brewer’s spent grains (BSG) proteins. The effects of extraction pH beyond the extraction yield, however, has not been investigated before. The present work examined the effects of extraction pH (pH 8–12) on BSG proteins’ (1) amino acid compositions, (2) secondary structures, (3) thermal stability, and (4) functionalities (i.e., water/oil holding capacity, emulsifying, and foaming properties). The ideal extraction temperature (60 °C) and BSG-to-solvent ratio (1:20 *w*/*v*) for maximizing the extraction yield were first determined to set the conditions for the pH effect study. The results showed that a higher extraction pH led to more balanced compositions between hydrophilic and hydrophobic amino acids and higher proportions of random coils structures indicating increased protein unfolding. This led to superior emulsifying properties of the extracted proteins with more than twofold improvement between pH 8 and a pH larger than 10. The extraction pH, nevertheless, had minimal impact on the water/oil holding capacity, foaming properties, and thermal denaturation propensity of the proteins. The present work demonstrated that a high alkaline pH at pH 11–12 was indeed ideal for both maximizing the extraction yield (37–46 wt.%) and proteins’ functionalities.

## 1. Introduction

Brewer’s spent grain (BSG) represents the most abundant side stream in beer brewing process where approximately 20 kg of BSG is produced per 100 L of beer [1]. An estimated 40 million tons of BSG are generated annually by the brewing industry globally to be primarily utilized as animal feeds, biogas production, or composting [2]. Not surprisingly, valorization of this substantial amount of BSG side stream—which are rich in fibers, proteins, and lipids—to valuable food products has been an active area of research [3]. In particular, BSG, which typically contains 15–30 wt.% proteins on a dry basis [4], holds a significant potential as an alternative protein source to address the increasing demand for proteins caused by the rising global population. Protein extraction from BSG is typically performed by alkaline extraction, solvent extraction, acid/salt extraction, or enzymatic hydrolysis [4]. Among these techniques, alkaline extraction is the most commonly used, mainly owing to its cost-effectiveness while maintaining a high extraction efficiency [5]. Isoelectric precipitation at an acidic pH has typically been employed to isolate BSG proteins after alkaline extraction.

Recently, a few studies have carried out optimization of the process variables in alkaline extraction (e.g., pH, BSG-to-solvent ratio, temperature, extraction time, and agitation rate) aimed at maximizing the protein extraction yield [6,7]. For BSG protein extracts obtained at the optimal yield, Silva et al. [7] proceeded to examine the proteins’ functional properties that were relevant to food applications. (e.g., solubility, water/oil holding capacity, emulsifying/foaming properties). Their results showed that BSG protein extracts obtained at the optimal yield generally did not exhibit the best functional properties. In addition to basic alkaline extraction, ultrasound-assisted alkaline extraction [8], high-pressure alkaline extraction [9], sequential alkaline extractions at different pHs [5], and simultaneous applications of high pressure, ultrasound, and BSG pre-treatment with acid [10] have also been proposed to maximize the extraction yield. 

Aside from protein functional properties, the effects of alkaline extraction processes on other properties of the BSG protein extracts have not been examined before. For instance, pH was identified in Silva et al. [7] as the governing process variable in alkaline extraction. Nevertheless, the effects of extraction pH on amino acid compositions of the BSG protein extracts, which greatly influence their nutritional quality, digestibility, sensory attributes, and functional properties [11] have not been investigated. Similarly, the effects of extraction pH on protein secondary structures and thermal stability have not been examined either. 

For this reason, the primary objective of the present work was to investigate the effects of extraction pH (i.e., pH 8 to 12) on the amino acid compositions, protein secondary structures, and thermal stability of BSG protein extracts. The insights gained from this study were then utilized to elucidate the trends observed in the effects of pH on the BSG protein extracts’ functionalities, namely (i) water/oil holding capacity, (ii) emulsifying, and (iii) foaming properties. The pH effect study was conducted at the ideal temperature and BSG-to-solvent ratio at a fixed extraction time. Herein, the ideal temperature and BSG-to-solvent ratio were defined as the values that led to the highest extraction yield. 

To this end, prior to the pH effect study, for each extraction pH, we investigated the effects of extraction temperature (i.e., 50 °C to 70 °C) and BSG-to-solvent ratio (i.e., 1:10 to 1:20 *w*/*v*) on the extraction yield. Notably, this study also aimed to validate the findings of Silva et al. [7] regarding the ideal conditions of pH, temperature, and BSG-to-solvent ratio to maximize the extraction yield of BSG proteins, while using raw BSG of different compositions (e.g., proteins, lipids, and carbohydrates) than that used in Silva et al. [7].

## 2. Results and Discussion

### 2.1. Ideal Conditions of Temperature and BSG-to-Solvent Ratio

**Figure 1:**
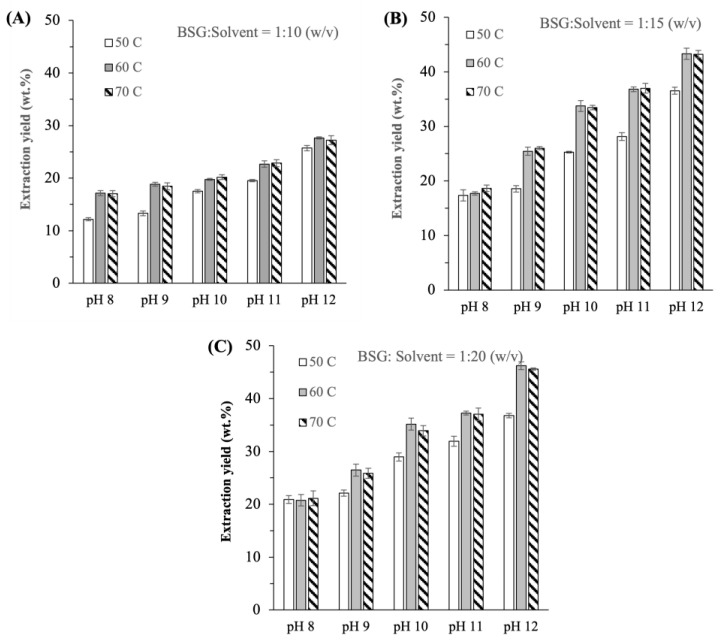
Effects of temperature on the extraction yield at different pHs for BSG-to-solvent ratios equal to (**A**) 1:10; (**B**) 1:15; (**C**) 1:20 (*w*/*v*).

For each extraction pH investigated (i.e., pH 8, 9, 10, 11, and 12), nine extraction conditions of varying temperatures (50, 60, and 70 °C) and BSG-to-solvent ratios (1:10, 1:15, and 1:20 *w*/*v*) were studied. For each extraction pH investigated, the extraction yield was found to increase as the BSG-to-solvent ratio was raised from 1:10 (*w*/*v*) (Figure 1A) to 1:15 (*w*/*v*) (Figure 1B), owing to more solvent molecules being available to interact with the protein molecules, resulting in the latter’s solubilization. Specifically, as the BSG-to-solvent ratio was raised from 1:10 to 1:15 (*w*/*v*), the percentage increase in the extraction yields at 50 °C, 60 °C, and 70 °C ranged from approximately 40 to 45%, 30 to 70%, and 10 to 60%, respectively, depending on the extraction pH. In this regard, the percentage increase in the extraction yield tended to be larger at a higher extraction pH. 

A further increase in the BSG-to-solvent ratio from 1:15 to 1:20 (*w*/*v*), nevertheless, led to a much smaller percentage increase (<20%) in the extraction yields (Figure 1C), suggesting that most of the soluble fraction of the proteins had already been extracted. Statistical analysis of the results obtained at BSG-to-solvent ratios of 1:15 and 1:20 (*w*/*v*) by Student’s t-test showed that the variations in the extraction yields between the two BSG-to-solvent ratios were statistically significant at the lowest and highest pHs investigated (i.e., pH 8 and 12, respectively) with p values of 0.009 and 0.016, respectively. Hence, from the point of view of maximizing the extraction yield, 1:20 (*w*/*v*) was determined as the ideal BSG-to-solvent ratio at which the extraction yields were in the range of ≈20 to 50 wt.%, depending on the temperature and pH. 

At the ideal BSG-to-solvent ratio (i.e., 1:20 *w*/*v*), the extraction yields at 60 °C were observed to be higher by roughly 20–25% compared to at 50 °C, particularly at pH ≥ 10 (Figure 1C). The higher extraction yields at 60 °C were attributed to higher protein solubility at higher solvent temperature, resulting in a larger mass transfer driving force of the proteins from the grains to the solution. Similar extraction yields were, nevertheless, observed at 60 °C and 70 °C. Higher extraction temperatures were not studied as they likely led to denaturation of the BSG proteins as discussed later in Section 2.2.4. Therefore, the ideal extraction temperature was determined to be at 60 °C. 

At the ideal temperature and BSG-to-solvent ratio, the extraction yields were determined to be equal to 20.8 ± 1.1, 26.5 ± 1.1, 35.2 ± 1.1, 37.3 ± 0.4, and 46.2 ± 0.7 wt.% at pH 8, 9, 10, 11, and 12, respectively. At these extraction yields, the amounts of proteins extracted per 100 g of lyophilized BSG ranged from ≈5.2 g at pH 8 to ≈11.7 g at pH 12 (Table 1). It was worth mentioning that the extraction yields reported in the present work were comparable in magnitude to the range of values reported in previous studies (≈10–60%) when similar alkaline extraction conditions were used [6,8,9]. 

Notably, higher extraction yields were observed with increasing extraction pH, which were also observed in Silva et al. [7]. The same trend had been observed in protein extractions from different food sources (e.g., lentil and tea) [12,13]. As the pH was increased above the isoelectric point (pI) of the protein—which for BSG protein, the pI was between ≈4 and 6 due to the presence of different proteins in BSG (e.g., hordeins and glutelins) [4]—the protein molecules became more negatively charged as more amino acid chains became deprotonated. The increased charges resulted in higher protein solubility and consequently higher extraction yields. A further increase in the extraction pH, however, likely led to significant protein denaturation, hence it was not pursued in the present work. 

Therefore, as far as extraction yield was concerned, the ideal extraction condition was determined to be at 1:20 (*w*/*v*) BSG-to-solvent ratio, 60 °C, and pH 12 with roughly 46 wt.% yield. This ideal condition was similar to the ideal condition determined in Silva et al. [7], namely 1:17 (*w*/*v*) BSG-to-solvent ratio, 60 °C, and pH 11. Silva et al. [7], however, reported a higher extraction yield of 87 wt.% because their yield was determined from the liquid extract prior to the isoelectric precipitation step, whereas in the present work, the yield was determined from the LPE obtained after the isoelectric precipitation step. Next, the physicochemical properties of BSG proteins extracted at different pHs were examined to determine the ideal extraction pH that considered not only the extraction yield, but also amino acid compositions, thermal stability, and functionalities. 

### 2.2. Effects of Extraction pH on BSG Proteins’ Physicochemical Properties

#### 2.2.1. Molecular Weight by Gel Electrophoresis

**Figure 2:**
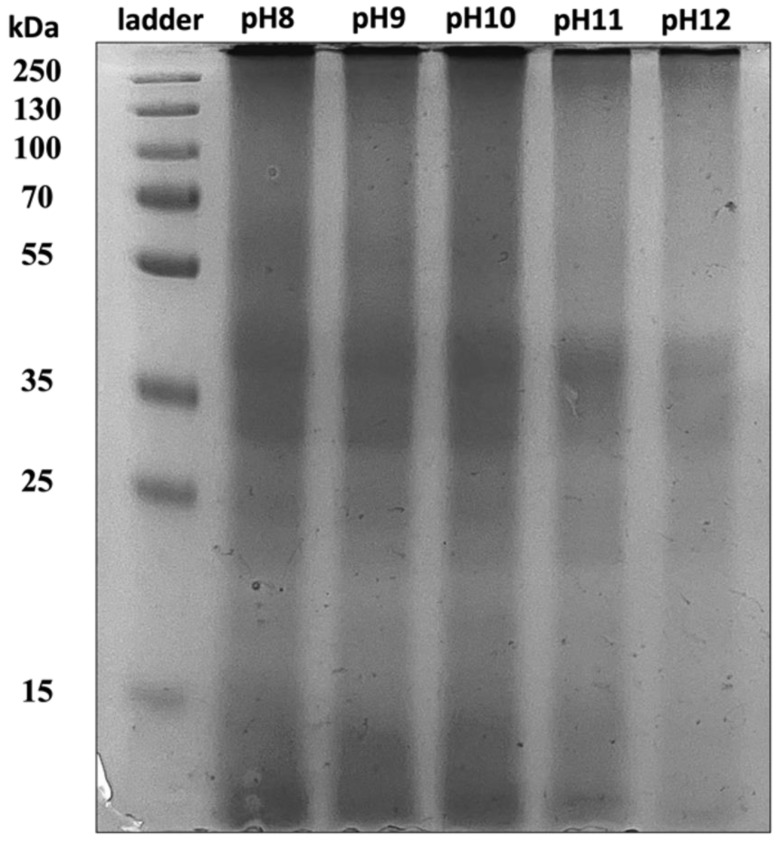
Gel electrophoresis image of the BSG proteins extracted at different pHs.

The SDS-PAGE gel image of the BSG proteins extracted at different pHs in Figure 2 shows the appearance of major bands at a molecular weight around 30–50 kDa and lighter intensity bands at around 55–80 kDa. The bands around 30–50 kDa and 55–80 kDa corresponded to the B-hordein and C-hordein proteins, respectively, with the B-hordein and C-hordein typically accounted for approximately 70–80% and 10–20% of the total hordeins in BSG, respectively [14]. Light-intensity bands at low molecular weights 15–25 kDa were also evident, attributed to A-hordein and/or polypeptide fragments of the proteins [15]. Importantly, the present SDS-PAGE results were in accordance with the previously reported gel electrophoresis results of BSG protein extracts [8,16,17]. Noticeably, the intensities of the 55–80 kDa and 15–25 kDa protein bands appeared to be slightly lower for proteins extracted at pH > 10 (≈10–15% lower as calculated by the ImageJ software 1.54a), suggesting that the A-hordein and C-hordein might undergo more folding leading to denaturation when extracted at higher alkaline pH.

#### 2.2.2. Amino Acid Compositions

Amino acid compositions of the BSG proteins extracted at different pHs were presented in Table 2. In general, the five most abundant amino acids present in the BSG protein extracts were glutamic acids (≈14–17 wt.%), proline (≈7–8 wt.%), tyrosine (≈8 wt.%), aspartic acid (≈7–8 wt.%), and depending on the extraction pH, either lysine, leucine, or phenylalanine (≈7–8 wt.%). Glutamic acid and proline were abundantly present in hordeins that made up most of the proteins in BSG [15], hence their significant compositions in BSG protein extracts were well-expected, and in agreement with previous results reported in [15,18]. The glutamic acid composition was found to decrease with increasing extraction pH from ≈17 wt.% at pH 8 to ≈14–15 wt.% at pH > 8 (*p* < 0.05). For each amino acid, the statistical significance of the effects of pH on its composition was characterized by the p-value presented in the last column of Table 2. A similar trend was observed for the proline composition that gradually decreased from ≈7.7 wt.% at pH 8 to ≈6.6 wt.% at pH 12 (*p* < 0.05). The decrease in the glutamic acid composition was accompanied by a slight increase in the leucine composition from ≈5.6 wt.% at pH 8 to ≈7.5 wt.% at pH ≥ 10 (*p* < 0.05). The higher composition of leucine as one of the essential amino acids observed at a higher extraction pH boded well for the nutritional quality of the BSG protein extracts. 

**Figure 3:**
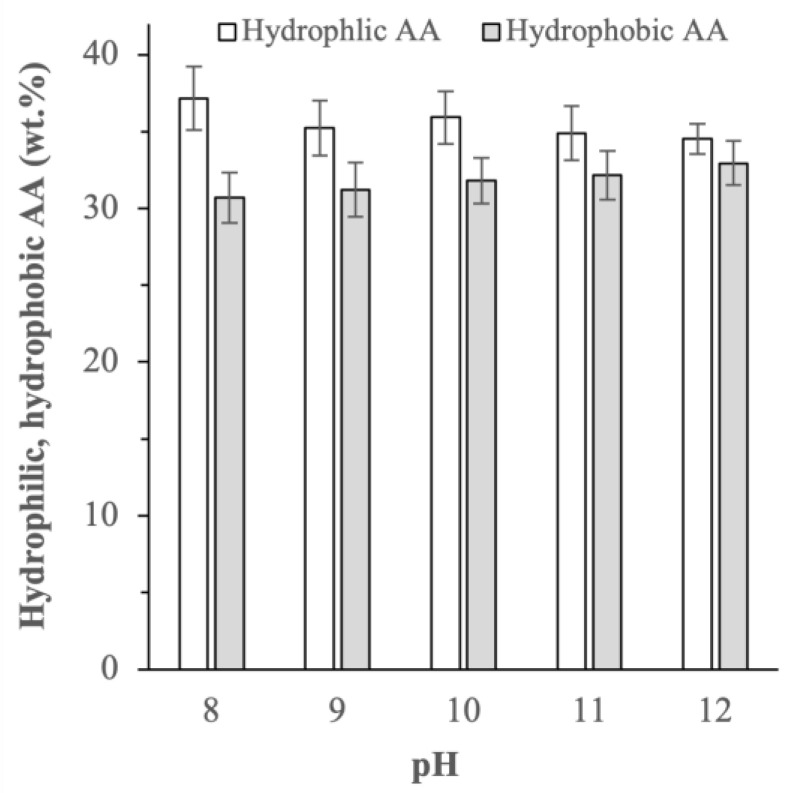
Hydrophilic and hydrophobic amino acid (AA) compositions of the BSG proteins extracted at different pHs (p-values of the difference in the hydrophilic and hydophobic AA compositions at extraction pH 8, 9, 10, 11, and 12 were equal to 0.031, 0.044, 0.231, 0.667, and 0.710, respectively).

At a higher extraction pH, the mass ratios of hydrophilic to hydrophobic amino acids decreased from ≈1.21 at pH 8 to ≈1.12 and 1.04 at pH 10 and 12, respectively (Figure 3). More specifically, the protein extracted at pH 8 contained approximately 37 wt.% hydrophilic and 30 wt.% hydrophobic amino acids *(*p < 0.05), whereas the protein extracted at pH 12 contained roughly equal compositions of hydrophilic and hydrophobic amino acids at about 33–34 wt.% (p > 0.05). The more balanced compositions between hydrophilic and hydrophobic amino acids in the proteins obtained at a higher extraction pH might influence their emulsifying properties as discussed later in Section 2.3.2.

#### 2.2.3. Protein Secondary Structures

Secondary structures of the BSG proteins extracted at different pHs were predominantly made up of β-turns (≈40–50%), followed by β-sheets (≈30%), α-helices (≈8–12%), and random coils (≈10–16%) (Table 3). The protein secondary structures were found to be influenced by the extraction pH. Specifically, the proportion of the β-turn structures gradually decreased with increasing pH from ≈52% at pH 8 to ≈43% at pH 12 (p < 0.05). Conversely, the proportions of α-helix and/or random-coil structures increased from ≈8% to ≈11–12% and from ≈10% to ≈16%, respectively, as the pH was increased from 8 to 12 (p < 0.05). On the other hand, the variations in the proportion of the β-sheet structures at different extraction pHs were found to be statistically insignificant (p > 0.05). 

The higher proportion of the random coils observed at higher extraction pHs signified increased protein unfolding, which was also indicated by the SDS-PAGE results. The protein unfolding could be attributed to the higher net negative charges possessed by protein molecules when they were extracted at higher alkaline pH. The larger net charges led to increased repulsive forces between the deprotonated amino acid chains in the protein, which in turn triggered protein unfolding [19]. In addition, intramolecular hydrogen bonds within the protein molecules, which contributed to the stability of the protein secondary structures, were disrupted at a higher alkaline pH mainly due to deprotonation of the amino groups, resulting in reduced numbers of hydrogen bond donors and acceptors. As a result, the protein molecules extracted at a higher alkaline pH exhibited a higher unfolding tendency [12].

#### 2.2.4. Thermal Stability

**Figure 4:**
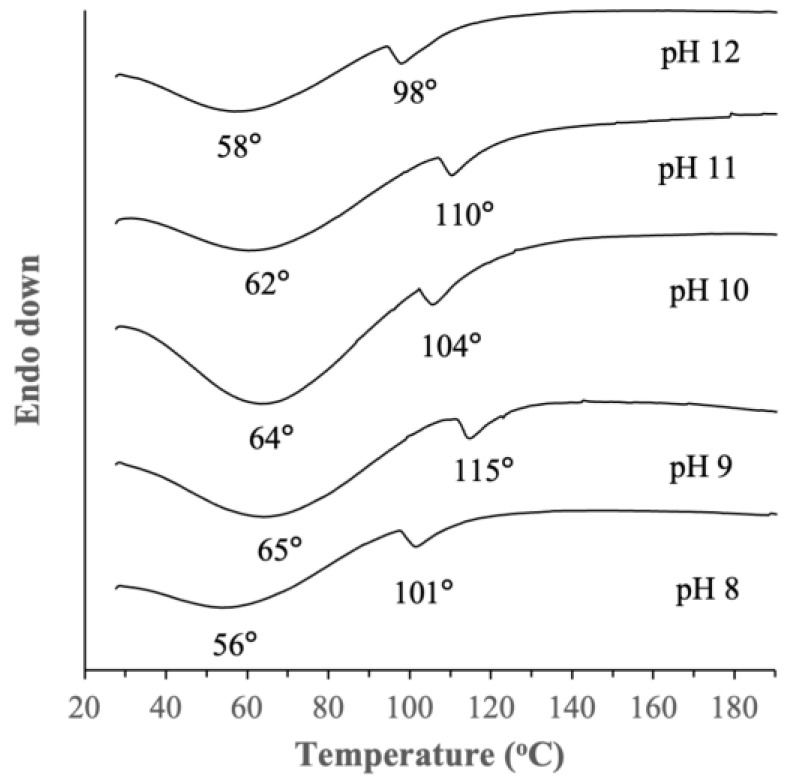
Differential scanning calorimetry (DSC) thermographs of BSG proteins extracted at different pHs.

DSC thermographs of the BSG proteins extracted at different pHs in Figure 4 showed the appearance of broad endothermic peaks centered around 56–65 °C signifying a transition event ($T_{t1}$) typically attributed to protein unfolding upon heating, which eventually led to protein denaturation. Upon further heating, a smaller endothermic peak centered at around 98–115 °C ($T_{t2}$) was observed which could be attributed to the loss of residual water of the protein molecules and/or complete denaturation of the proteins [20]. To the best of our knowledge, thermal stability of BSG protein extracts has not been reported before. On this note, a previous study, which performed DSC of dried raw BSG, reported a similar endothermic event at around 59 °C [21]. The effects of extraction pH on the onset of protein denaturation appeared to be random, with no specific trend. The proteins extracted at pH 8 and 12 exhibited lower $T_{t1}$ at 56–58 °C compared to 62–65 °C for the proteins extracted at pH 9–11, indicating the latter’s slightly higher thermal stability. A similar observation was made for $T_{t2}$. The difference in their thermal stability could be attributed to the variations in the molecular weight distribution of the proteins, amino acid compositions, and protein secondary structures as previously discussed. 

### 2.3. Effects of Extraction pH on BSG Proteins’ Functional Properties

#### 2.3.1. Water and Oil Holding Capacity

**Figure 5:**
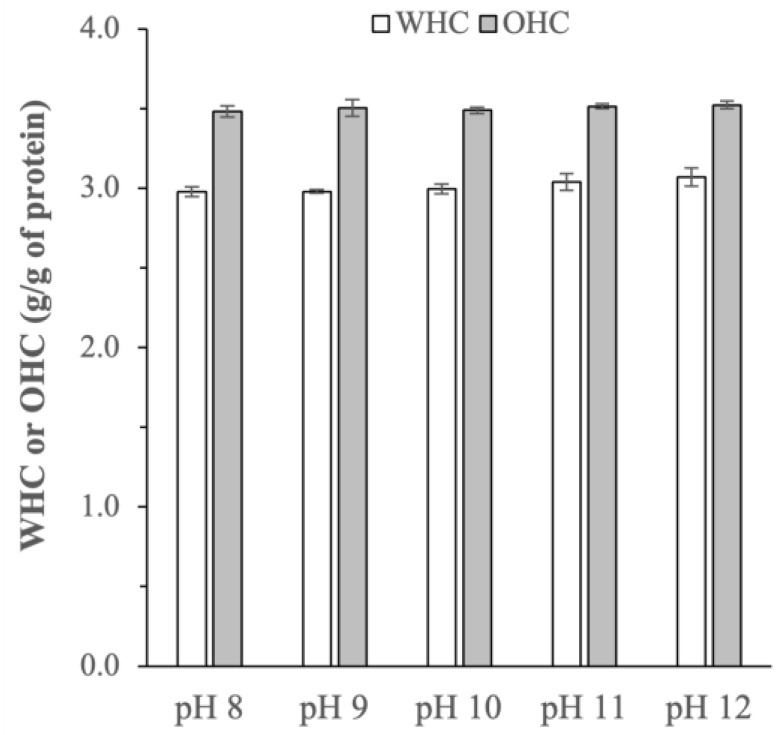
Water holding capacity (WHC) and oil holding capacity (OHC) of BSG proteins extracted at different pHs (p-values for WHC and OHC of the protein extracts obtained at different extraction pHs were equal to 0.062 and 0.563, respectively).

The water holding capacity (WHC) of proteins refers to their ability to retain and absorb water, whereas oil holding capacity (OHC) characterizes their oil-binding activity. Both properties are important indices in food applications as WHC reflects the viscosity and textures of the proteins, while OHC influences the proteins’ thickening and emulsifying properties [22]. The WHC and OHC of the BSG protein extracts were determined to be between 2.98 ± 0.03 to 3.07 ± 0.05 g/g of protein and 3.48 ± 0.03 to 3.52 ± 0.02 g/g of protein, respectively (Figure 5). The WHC and OHC reported in the present work were comparable in magnitude to the values reported previously in [7,8], as well as the WHC and OHC values of other alternative plant-based protein sources (e.g., legumes) [23]. 

The effects of extraction pH on the WHC and OHC were found to be minimal (p > 0.05), despite the pH’s influences on the molecular weight distributions, amino acid compositions, and protein secondary structures as earlier discussed. Theoretically, a higher degree of protein unfolding observed at a higher extraction pH might lead to higher exposure of the protein’s water-binding sites, which in turn increases the protein’s polarity and consequently results in higher WHC [24]. Similarly, the increased proportion of hydrophobic amino acids observed at a higher extraction pH could promote greater interactions between proteins and lipids through the binding of aliphatic chains of the lipids with non-polar side chains of the amino acids, resulting in higher OHC [25]. Such increases in the WHC and OHC, however, were not observed in the present work, likely due to relatively small changes in the amino acid composition and protein conformation as the extraction pH was varied. 

#### 2.3.2. Emulsifying Activity and Stability Indexes

**Figure 6:**
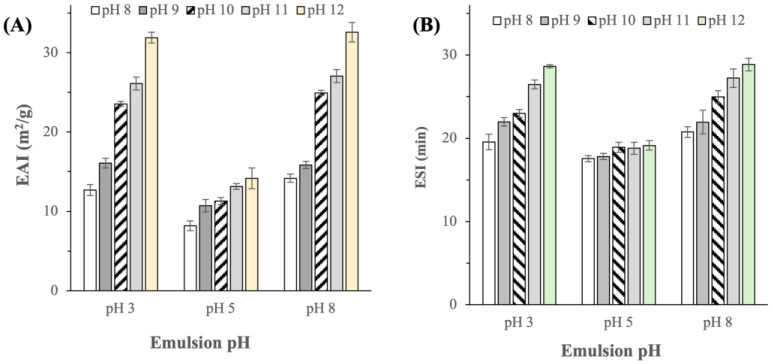
(**A**) Emulsifying activity index (EAI) and (**B**) emulsifying stability index (ESI) of BSG protein extracted at different pHs (p-values for EAI of the protein extracts obtained at different extraction pHs were all equal to <0.0005 in emulsions of pH 3, 5, and 8; p-values for ESI of the protein extracts obtained at different extraction pHs were equal to <0.0005, 0.016, and <0.0005, in emulsions of pH 3, 5, and 8, respectively).

Proteins represent the most commonly used stabilizers in food emulsions [26]. Therefore, the abilities of the BSG proteins extracted at different pHs to form and stabilize emulsions were characterized by the emulsifying activity index (EAI) and emulsifying stability index (ESI), respectively. EAI and ESI were characterized at three different pH levels of the emulsions (i.e., pH 3, 5, and 8). In emulsions with pH 3 and pH 8, the EAI values of the protein extracts were found to be similar in magnitude, ranging from ≈12 to 32 m^2^/g depending on the extraction pH (Figure 6A). Similar ESI values were also observed in emulsions with pH 3 and pH 8, where the ESI ranged from ≈20 to 28 min depending on the extraction pH (Figure 6B). 

On the other hand, in emulsions with pH 5, EAI and ESI were found to be considerably lower at 8–14 m^2^/g and 17–19 min, respectively (Figure 6). At pH 5, which was at or near to the typical pI of BSG proteins [4], protein molecules exhibited a nearly zero net charge, resulting in their poor solubility. Thereby, only a small fraction of the BSG protein extract could dissolve in the water/oil solution and subsequently diffuse to the oil–water interface to stabilize the emulsified droplets. Furthermore, the absence of a net protein charge caused a void of electrostatic repulsive forces at the oil–water interface, which in turn increased the propensity of the emulsified droplets to aggregate, hence destabilizing the emulsion [27]. 

Significantly, BSG proteins extracted at a higher pH were found to exhibit higher EAI and ESI values indicating their superior emulsifying properties (p  < 0.05), particularly in emulsions with pH 3 and 8. The EAI of proteins extracted at pH > 10 was higher by more than twofold compared to the EAI of proteins extracted at pH 8. The higher EAI and ESI observed at a higher extraction pH could be attributed to the more balanced compositions between hydrophilic and hydrophobic amino acids at higher extraction pH, allowing the protein molecules to better orient themselves at the oil–water interface to reduce the interfacial tension, resulting in stable emulsions [28]. Importantly, the reported EAI and ESI were in the same order of magnitude, albeit at slightly lower values, compared to the EAI and ESI values previously reported in [7,8]

#### 2.3.3. Foaming Capacity and Stability

**Figure 7:**
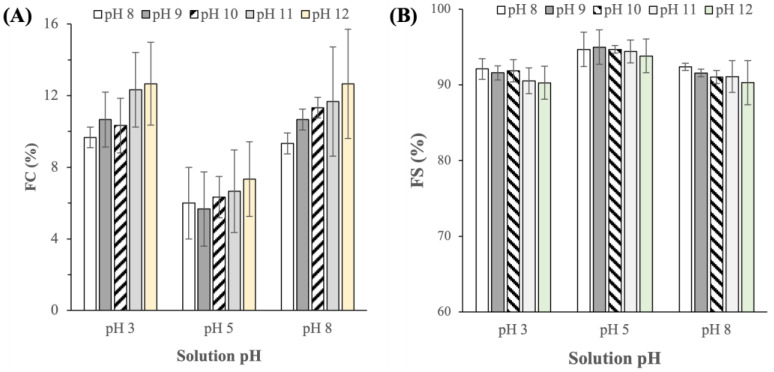
(**A**) Foaming capacity (FC) and (**B**) foaming stability (FS) of BSG proteins extracted at different pHs (p-values for FC of the protein extracts obtained at different extraction pHs were equal to 0.217, 0.858, and 0.380 in emulsions of pH 3, 5, and 8, respectively; p-values for FS of the protein extracts obtained at different extraction pHs were equal to 0.546, 0.953, and 0.650, in emulsions of pH 3, 5, and 8, respectively).

In addition to their roles as emulsifiers, proteins are also prominent foaming agents in the food industry [29]. The abilities of the BSG proteins extracted at different pHs to form and stabilize foams were characterized by the foam capacity (FC) and foam stability (FS), respectively. Similar to the emulsifying properties, the foaming properties were characterized at three different pH levels of the solution (i.e., pH 3, 5, and 8). The results showed that the BSG protein extracts exhibited FC in the range of ≈6 to 13% (Figure 7A), which were comparable to the value reported in Li et al. [8] and were also comparable to the FCs of other plant-based alternative proteins, such as soy bean, faba bean, and rice grain proteins [30,31,32]. Importantly, the foams exhibited good stability as evidenced by FS > 90% after 30 min (Figure 7B). 

Similar to the trend in the EAI results, the FCs were observed to be higher in solutions with pH 3 and 8 (FC ≈ 9–13%) compared to solutions with pH 5 (FC ≈ 6–7%). The lower FC in solutions with pH 5 could also be attributed to the low protein solubility at a pH near its pI, which limits the amount of protein molecules available at the air–water interface to stabilize the trapped air molecules. Unlike the trend in EAI, however, the effects of the extraction pH on the FC and FI were not found to be statistically significant (p > 0.05).

## 3. Materials and Methods

### 3.1. Materials

Lyophilized BSG (23 ± 0.5 g protein/100 g of BSG) stored in polyethylene bags at −80 °C was generously donated by Asia Pacific Breweries (Singapore) Pte. Ltd. Besides proteins, the lyophilized BSG contained roughly 64 ± 3 g carbohydrates, 9 ± 0.1 g lipids, and 4 ± 0.05 g ash per 100 g of BSG [33]. Chemicals used for gel electrophoresis, i.e., 30% Acrylamide/Bis-acrylamide (29:1 *w*/*w*), tris, sodium dodecyl sulfate (SDS), ammonium persulfate, *N*,*N*,*N*′,*N*′-Tetramethylethylenediamine (TEMED), ß-mercaptoethanol, Coomassie Brilliant Blue G-250, and sodium dodecyl sulfate-polyacrylamide gel (SDS-PAGE) sample loading buffer were purchased from Bio-Rad (Singapore). A PageRuler™ pre-stained protein ladder (15 to 250 kDa) and corn oil were purchased from Thermo Scientific (Singapore). Analytical-grade NaOH, HCl, acetonitrile, Bradford reagent, amino acid standard (AAS18), bovine serum albumin (BSA), *N*-*tert*-Butyldimethylsilyl-*N*-methyltrifluoroacetamide (MTBSTFA), and *tert*-Butyldimethylchlorosilane (TBDMSCI) were purchased from Sigma-Aldrich (Singapore).

### 3.2. Methods

#### 3.2.1. Extraction and Isolation of BSG Proteins

Alkaline extraction of BSG proteins was performed in triplicate at five different pHs (i.e., pH 8, 9, 10, 11, and 12) using diluted aqueous NaOH solution as the solvent. For each pH level, the extraction was performed at three BSG-to-solvent ratios (i.e., 1:10, 1:15, and 1:20 *w*/*v*) and temperatures (i.e., 50, 60, and 70 °C), totalling nine extraction experiments at each pH. The ranges of the BSG-to-solvent ratios and temperatures investigated were determined based on the typical values used in previous studies on alkaline extraction of BSG proteins [18,34]. Briefly, the lyophilized BSG was thawed at room temperature for 3 h, after which it was dispersed in a conical flask containing NaOH solution of different pHs and placed in a shaking incubator at 250 rpm. After 2 h incubation in NaOH, the BSG suspension was centrifuged at 11,000× *g* for 20 min, and the supernatant containing the extracted proteins was collected. 

**Figure A1:**
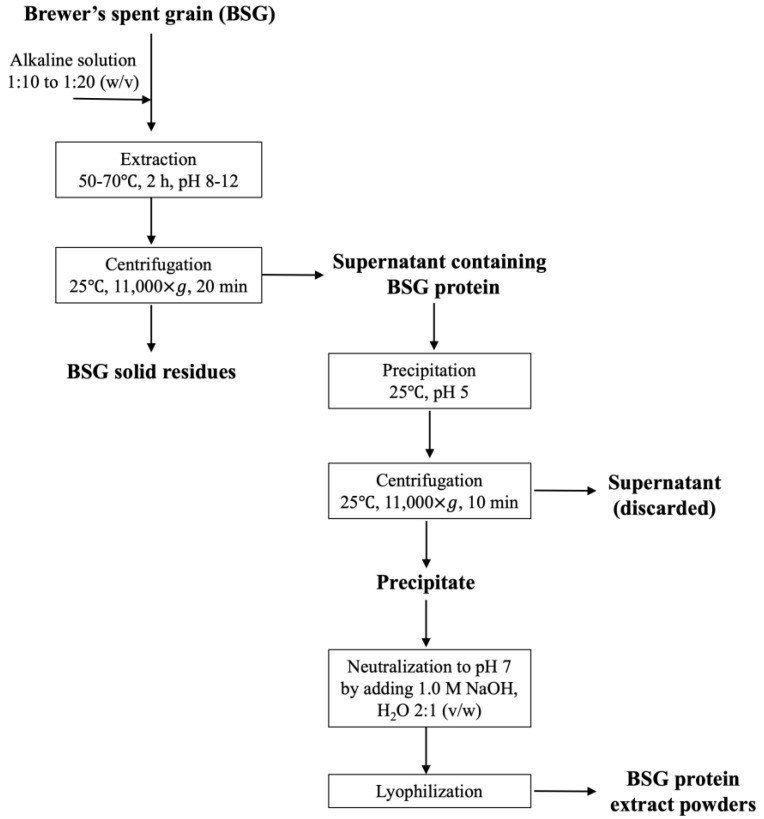
Flowchart of the alkaline extraction–isoelectric precipitation process used to extract and isolate proteins from lyophilized raw BSG.

Next, the protein extract in the supernatant was solidified by isoelectric precipitation by lowering the pH of the supernatant to 5.0 with an addition of 1.0 N HCl. The resultant suspension was then centrifuged at 11,000× *g* for 10 min, after which the sediment containing the BSG protein extract was collected and re-suspended in water. The pH of the protein extract solution (PES) was adjusted to 7.0 by adding 1.0 M NaOH, followed by 24 h lyophilization (Alpha 1–2 LD Plus freeze dryer, Martin Christ, Osterode am Harz, Germany) at −52 °C and 0.05 mbar. The lyophilized protein extract (LPE) was stored at −20 °C until further analysis. A flowchart depicting the alkali extraction–isoelectric precipitation of the lyophilized BSG is shown in Figure A1 of Appendix A.

#### 3.2.2. Extraction Yield and Extracted Protein Content

The extraction yield was defined in Equation (1) as the ratio of the mass of protein recovered from the PES to the total mass of protein in the lyophilized BSG. The mass of protein in the PES was determined in triplicate by the Bradford assay using BSA as the protein standard. Briefly, 0.2 mL of diluted PES and 1.8 mL of Bradford solution were mixed and allowed to sit for 5 min at room temperature. Afterwards, the absorbance of the mixed sample was measured at a wavelength of 595 nm by a UV–Vis spectrophotometer (UV Mini-1240, Shimadzu, Kyoto, Japan). The extracted protein content was calculated from the mass of protein recovered from the PES (g) per 100 g of lyophilized BSG.

(1) $$Extraction\;yield=\frac{Mass\;of\;protein\;in\;the\;PES}{Total\;mass\;of\;protein\;in\;the\;lyophilized\;BSG}\times 100\%$$

#### 3.2.3. Gel Electrophoresis

SDS-PAGE was performed in triplicate under a reducing condition in a vertical mini-gel electrophoresis unit (BioRad, Singapore). The SDS-PAGE gel was prepared with 12% (*w*/*v*) acrylamide separating gel and 4% (*w*/*v*) acrylamide stacking gel. Briefly, the LPE was dispersed in deionized water at 15 mg/mL and mixed with an equal volume of 2× SDS-PAGE sample loading buffer containing 5% (*v*/*v*) ß-mercaptoethanol, followed by 5 min incubation at 95 °C. Afterward, the protein extract solution was centrifuged at 11,000× *g* for 5 min, and 10 μL of the supernatant was loaded onto the gel. The gel electrophoresis was performed at 90 V for 30 min, after which the voltage was increased to 110 V for the next 90 min. Afterwards, the gel was stained with Coomassie Brilliant Blue G-250 for 1 h and then de-stained with deionized water for another 1 h. Images of the stained protein bands were visualized and taken by a gel documentation system (GeneSys, Frederick, MD, USA).

#### 3.2.4. Amino Acid Compositions

Amino acid compositions of the BSG protein extracts were determined in triplicate by gas chromatography–mass spectrometry (GC-MS) using 7890A and 5975C inert MSD with a Triple Axis Detector (Agilent Technologies, Santa Clara, CA, USA), following the protocols described in [35]. Briefly, 5 mg of the LPE was hydrolyzed using 0.5 mL of 6 M HCl at 110 °C for 24 h. Afterwards, 100 μL of acetonitrile and 100 μL of MTBSTFA + 1% TBDMSCI were added for deproteinization and derivatization, respectively. The resultant solution was then incubated for 60 min at 100 °C under gentle stirring to promote the derivatization reaction. The amino acid content was then characterized by GC-MS using Agilent DB-5 column with amino acid standard as the reference. 

#### 3.2.5. Protein Secondary Structures 

Protein secondary structures of the BSG protein extracts were determined in triplicate by Fourier-transform infrared spectroscopy (FTIR) deconvolution, following the protocols described in [36]. The FTIR analysis was performed using a Thermo Scientific Nicolet™ iS50 FTIR Spectrometer (Thermo Fisher Scientific Inc., Waltham, MA, USA) between 4000 and 400 $cm^{-1}$ at 4 $cm^{-1}$ resolution. A second derivative analysis was performed on the amide I band data between 1600 and 1700 $cm^{-1}$ using Origin software 2019b (OriginLab Inc., Northampton, MA, USA) with the Gaussian curve fitting procedure. In accordance with [36], peaks between 1610 and 1642 $cm^{-1}$, 1643 and 1650 $cm^{-1}$, 1650 and 1659 $cm^{-1}$, and 1660 and 1699 $cm^{-1}$ were assigned as β-sheet, random-coil, α-helix, and β-turn secondary structures, respectively. The proportion of each secondary structure was calculated by dividing the area belonging to the said secondary structure to the total area of the deconvoluted spectra.

#### 3.2.6. Thermal Stability

Thermal stability of the BSG protein extracts was characterized by differential scanning calorimetry (DSC) using 822e CryoDSC (Mettler Toledo, Columbus, OH, USA). The DSC analysis was performed on the LPE at a heating rate of 10 °C/min from 25 °C to 200 °C under nitrogen atmosphere at a flow rate of 50 mL/min. 

#### 3.2.7. Water and Oil Holding Capacities

Water holding capacity (WHC) and oil holding capacity (OHC) of the BSG protein extracts were determined in triplicate following the protocols described in [8]. Briefly, distilled water or corn oil was added into a pre-weighed tube containing 10 mg of the LPE. The suspension was vortexed for 1 min and left to sit at room temperature for 30 min. The suspension was then centrifuged at 2800× *g* for 10 min, and the supernatant was discarded. Afterwards, the tube that contained water or oil-bounded proteins was weighed. The water and oil holding capacity were calculated using Equation (2) and expressed as grams of water/oil bound per gram of the protein extract.

(2) $$WHC\;or\;OHC\left(g/g\;of\;protein\right)=\frac{W_{2}-W_{1}}{W_{0}}$$

where $W_{2}$ is the mass of the tube containing the water/oil-bound proteins (g), $W_{1}$ is the initial mass of the tube containing the LPE, and $W_{0}$ is the mass of the LPE inside the tube.

#### 3.2.8. Emulsifying Properties

Emulsifying properties of the BSG protein extracts were characterized in triplicate by the emulsifying activity index (EAI) and emulsifying stability index (ESI) defined in Equations (3) and (4), respectively. Following the protocols described in [18], emulsions were prepared by mixing 15 mL of 1 wt.% aqueous protein extract solution and 5 mL of corn oil in a test tube. The pH of the emulsions was adjusted to pH 3, 5, and 8 by addition of HCl or NaOH. The mixtures were then homogenized using an ultrasonic homogenizer (CV 24 Vibra Cell, Sonics & Materials, Inc., Newton, CT, USA) for 3 min. Afterwards, 50 μL aliquot was pipetted from the bottom of the tubes at 0 and 10 min and subsequently was diluted with 2.5 mL of 0.1% (*w*/*v*) SDS solution. Absorbances of the diluted emulsions at 0 min ($A_{0}$) and 10 min ($A_{10}$) were measured at a wavelength of 500 nm using an UV–visible spectrophotometer. The EAI and ESI at different pHs were then calculated using Equations (3) and (4), respectively (∆t = 10 min).

(3) $$EAI(m^{2}/g)=\frac{2\times 2.303\times A_{0}}{0.25\times mass\;of\;protein\;extract\;(g)}$$

(4) $$ESI(min)=\frac{A_{0}\times ∆t}{A_{0}-A_{10}}$$

#### 3.2.9. Foaming Properties

Foaming properties of the BSG protein extracts were characterized in triplicate by the foaming capacity (FC) and foaming stability (FS) defined in Equations (5) and (6), respectively. Following the protocols described in [18], 10 mL ($V_{initial}$) of 0.5 wt.% aqueous protein extract solution was prepared at pH 3, 5, and 8, after which the solution was homogenized for 3 min at room temperature. The volume of the whipped solution was recorded at 0 min ($V_{0}$) and 30 min ($V_{30}$). FC and FS at different pHs were calculated using Equations (5) and (6), respectively.

(5) $$FC\left(\%\right)=\frac{V_{0}-V_{initial}}{V_{initial}}\times 100\%$$

(6) $$FS\left(\%\right)=\frac{V_{30}}{V_{0}}\times 100\%$$

#### 3.2.10. Statistical Analysis

The statistical significances of the characterization results (p≤ 0.05) were determined using Microsoft Excel by either Student’s t-test for the effects of BSG-to-solvent ratio or one-way analysis of variance (ANOVA) test (without post-hoc analysis) for the effects of the extraction pH. 

## 4. Conclusions

A high alkaline pH has been previously reported to improve the BSG protein extraction yield. The present work demonstrated that the extraction pH greatly influenced not only the extraction yield, but also the amino acid compositions and secondary structures of the extracted proteins. Specifically, a high extraction pH (pH 11–12) led to more balanced compositions between hydrophilic and hydrophobic amino acids, as well as increased proportions of leucine, which is an essential amino acid. The more balanced amino acid compositions led to superior emulsifying properties of the proteins, while the water/oil holding capacities and foaming properties were minimally affected. A higher extraction pH also led to a higher proportion of random-coil structures indicating increased protein unfolding that also influenced the proteins’ emulsifying properties through increased exposure of water-binding sites. The effects on extraction pH on the thermal stability was, nevertheless, minimal as evidenced by similar denaturation temperature. In summary, the present work showed that a high alkaline pH was indeed ideal for obtaining high extraction yield and better protein functionalities. The present work also reaffirmed the previous finding on the ideal extraction temperature and BSG-to-solvent ratio to maximize the extraction yield. Hence, future works in BSG protein extraction by the alkaline method can directly operate within the recommended parameters. Last but not least, the higher degree of protein unfolding observed at a high alkaline pH in the present work necessitates future investigations on the storage stability of BSG proteins extracted at high alkaline pH, which are currently undergoing in our laboratory. 

## Figures and Tables

**Table 1:** Extracted protein content at 60 °C and BSG-to-solvent ratio = 1:20 (*w*/*v*).

pH	8	9	10	11	12
Extracted protein content (g/100 g of lyophilized BSG)	5.2 ± 0.2	6.7 ± 0.3	8.9 ± 0.3	9.4 ± 0.1	11.7 ± 0.2

**Table 2:** Effects of pH on amino acid compositions (wt.%) ^.

Amino Acid Composition (wt.%)	pH 8	pH 9	pH 10	pH 11	pH 12	p-Value
L-Alanine	6.21 ± 0.01	6.32 ± 0.31	5.88 ± 0.01	5.83 ± 0.14	5.68 ± 0.33	0.018
Glycine	1.95 ± 0.40	2.82 ± 0.58	3.01 ± 0.47	2.84 ± 0.37	2.79 ± 0.17	0.074
L-Valine	4.48 ± 0.59	5.51 ± 0.36	5.49 ± 0.14	4.96 ± 0.10	4.80 ± 0.43	0.027
L-Leucine	5.61 ± 0.26	6.77 ± 0.29	***7.55*** ± ***0.19***	***7.61*** ± ***0.22***	7.51 ± 0.06	0.001
Isoleucine	4.99 ± 0.36	4.75 ± 0.57	4.61 ± 0.53	4.42 ± 0.52	4.70 ± 0.42	0.696
L-Threonine	5.04 ± 0.13	5.70 ± 0.13	5.44 ± 0.01	5.30 ± 0.08	5.09 ± 0.39	0.012
L-Proline	***7.70*** ± ***0.19***	***7.10*** ± ***0.28***	6.72 ± 0.13	6.83 ± 0.21	6.59 ± 0.17	0.002
L-Methionine	2.10 ± 0.12	1.71 ± 0.30	1.82 ± 0.57	1.94 ± 0.52	2.14 ± 0.29	0.648
L-Serine	4.42 ± 0.20	4.50 ± 0.15	4.19 ± 0.39	4.06 ± 0.26	4.04 ± 0.08	0.144
L-Phenylalanine	***7.31*** ± ***0.35***	6.15 ± 0.57	6.46 ± 0.02	***7.40*** ± ***0.27***	***8.11*** ± ***0.29***	<0.0005
L-Aspartic acid	***8.01*** ± ***0.14***	***7.14*** ± ***0.73***	***7.55*** ± ***0.37***	7.14 ± 0.63	***7.57*** ± ***0.21***	0.211
L-Glutamic acid	***17.40*** ± ***0.13***	***15.41*** ± ***0.20***	***14.63*** ± ***0.15***	***14.62*** ± ***0.17***	***14.33*** ± ***0.28***	<0.0005
L-Ornithine	5.69 ± 0.64	4.91 ± 0.22	5.70 ± 0.65	5.49 ± 0.55	5.10 ± 0.14	0.252
L-Lysine	6.05 ± 0.53	***7.78*** ± ***0.29***	***8.04*** ± ***0.21***	***7.66*** ± ***0.08***	***7.52*** ± ***0.16***	0.0005
L-Histidine	3.55 ± 0.02	3.30 ± 0.48	3.18 ± 0.52	3.97 ± 0.14	3.82 ± 0.32	0.092
L-Tyrosine	***8.32*** ± ***0.03***	***8.22*** ± ***0.17***	***7.66*** ± ***0.32***	***7.89*** ± ***0.37***	***8.13*** ± ***0.32***	0.078
L-Cystine	1.16 ± 0.18	1.90 ± 0.36	2.08 ± 0.62	2.05 ± 0.43	2.08 ± 0.19	0.068

^ The five most abundant amino acids at each extraction pH are highlighted in bold italics.

**Table 3:** Effects of extraction pH on protein secondary structures.

Assignment (%)	pH 8	pH 9	pH 10	pH 11	pH 12	p-Value
α-helix	8.4 ± 0.3	8.4 ± 0.4	10.4 ± 0.4	11.2 ± 0.4	11.8 ± 0.3	<0.0005
β-sheet	28.9 ± 1.2	29.8 ± 1.3	29.9 ± 1.1	30.7 ± 1.2	28.8 ± 0.7	0.281
β-turn	52.5 ± 2.2	49.1 ± 2.1	46.0 ± 1.6	48.0 ± 1.8	43.2 ± 1.1	0.001
Random coil	10.2 ± 0.4	12.7 ± 0.5	13.7 ± 0.5	10.1 ± 0.4	16.2 ± 0.4	<0.0005

## Data Availability

Data will be made available upon request.
